# Parental COVID‐19–related health information practises, sources, evaluations and needs: A qualitative interview study

**DOI:** 10.1111/hex.13688

**Published:** 2022-12-08

**Authors:** Hala Altawil, Ronny Klawunn, Marie‐Luise Dierks, Jonas Lander

**Affiliations:** ^1^ Deparment for Patient Orientation and Health Education, Institute for Epidemiology, Social Medicine and Health System Research Hannover Medical School (MHH) Hannover Germany

**Keywords:** child health, COVID‐19 health information, information behaviour, information handling

## Abstract

**Background:**

Parents of infants and young children may have specific health information needs and preferences, as they are responsible for their children's health. COVID‐19 posed many challenges for families, not least in terms of the constantly updated disease‐prevention guidelines. However, little is known about parents' experiences with this unprecedented situation, that is, how and where they seek, use and evaluate COVID‐19 (child)‐specific health information. We aimed to find out more about this to provide insights to health (information) providers when communicating pandemic information to parents.

**Methods:**

We conducted semistructured telephone interviews (August to October 2020) with a purposively selected sample of 20 German‐speaking and 10 Arabic‐speaking parents of children up to 4 years old. Recruitment occurred through multiple channels, including childcare institutions and social media. Qualitative content analysis of the interview transcripts illustrates the main differences between the two groups.

**Results:**

By the time the interviews were conducted (mid‐2020), some parents reported to seek information less actively or not at all, compared to the beginning of COVID‐19. German speakers frequently used Google to obtain information, whereas Arabic speakers mentioned social media (particularly Facebook) as a central source. However, medical providers were the most trusted source for child health. Though determining the credibility of online information was difficult for some parents, others, mostly German speakers (middle–high education), were aware of some author‐related criteria. When deciding on information use, parents often rely on their own judgement and gut instinct. Besides the necessity to disseminate information via multiple outlets to reach all parents, Arabic speakers desired audio‐visual and translation tools to facilitate understanding.

**Discussion and Public Conclusion:**

Apart from education, language and knowledge of the health system and of the attributes of credible information may determine its quality and consequent decisions. There seems to be a considerable need to foster knowledge about reliable information sources, a greater understanding of the range of quality criteria and specific support for nonnative speakers, not least to better inform parents' decision‐making.

**Patient and Public Contribution:**

A parent panel (*n* = 7) contributed to gathering ideas regarding recruitment, discussing initial results and the choice of topics and questions for a second interview phase.

## 1 INTRODUCTION

COVID‐19 has illuminated the role and relevance of health information (HI) more than ever before.^1^, ^2^, ^3^ While the situation affected everyone, the situation of, for instance, parents of infants and young children is special given their responsibility towards children when estimating the risks and impacts of everyday‐life activities on health.^4^, ^5^, ^6^

Given this, and also since many core information and consultation services were substantially burdened, seeking, understanding and applying HI to respond rapidly and properly may be more challenging.^7^ Though the number of parents searching the web for child health‐related information is high and rising,^8^, ^9^ the ability to handle (digital) HI depends, on the one hand, on individual health literacy (HL)—which has further worsened according to recent representative statistics for Germany, showing a particular deficit for digital HL, and variations among population groups.^10^ On the other hand, healthcare organizations have a key role in actively supporting individuals' information and decision processes,^11^ particularly amid the torrent of information including inaccurate and false ones.^12^ Further, (digital) HI often disregards its target populations' specific needs^13^ and instead, parents may draw their information from various sources,^14^ such as health professionals (HPs) and peers.

In addition, at least four aspects specific to parental COVID‐19 information behaviour (IB) can be identified. First, empirical insights into parental HI behaviour—for instance, regarding prevention measures—would reveal whether parents sought information generally in terms of ‘COVID‐19’ or specifically regarding infection prevention for the child. Second, aspects related to (mis)trust, acceptance of behavioural advice and handling uncertainty are decisive for health‐related decision‐making.^15^ Respective factors, however, still need to be understood, as few comparable situations have been studied. This is particularly true for parents, as child health is a highly emotional topic.^12^ Understanding parents' IB may be relevant for regular, postpandemic HI issues, as the amount of available digital information is constantly growing.

Third, prior research suggests that user perspectives are often inadequately considered for digital HI.^16^, ^17^ Parents' information requires particular attention, as they need to make decisions on behalf of their children (and family).^18^ Last, it can be vital to clarify possible differences among culturally and linguistically diverse user groups: While all societal groups should have equal information access, insufficient language proficiency in the host country may limit information access for migrant groups.^19^ Moreover, sociocultural backgrounds may substantially affect how individuals apply advice^20^; the case of parents may be an exemplar.

A study on the differences in the perceived risk perceptions between nine ethnic minority groups of young adults aged 24–26 in Germany found, contrary to expectations, a higher increase in their COVID‐19‐related health risk perceptions in comparison with the general population and could not explain many of the few ethnic differences discovered.^21^ Given the need for a deeper understanding about potential differences for parents with distinct social and cultural backgrounds, the target group of this study is split up into (a) parents with German as native or second language, affected and not affected by COVID‐19, and (b) migrant parents, affected and not‐affected by COVID‐19, and represented by those who migrated from the Arab region recently. The latter is characterized by a small but growing population in Germany, particularly since 2015.

The specific objectives of this study were to explore how parents:

(1) access and search for COVID‐19–related (child) HI,
(2) understand and appraise COVID‐19–related (child) HI to make decisions, handle challenges and how they trust/distrust certain sources,
(3) apply COVID‐19–related (child) HI in daily life,
(4) express needs and preferences regarding the provision and communication of (digital) HI, and
(5) differs due to cultural and/or linguistic backgrounds regarding objectives 1–4.

## 2 MATERIALS AND METHODS

This study employed an exploratory, qualitative interview design. Its development, conduct, analysis and reporting were performed in line with the Consolidated Criteria for Reporting Qualitative Research (COREQ) Checklist^22^ (Supporting Information: Appendix 1).

### 2.1 Sampling and target group

To gather a broad spectrum of parental views, we aimed to increase the diversity of the ex‐ante identified sample via purposive and snowball sampling, according to gender, number and age of children and education status for two groups: (native) German‐speaking residents (hereafter referred to as German speakers, *n* = 20), and native Arabic‐speaking migrants—particularly those who migrated to Germany during the last few years (hereafter referred to as Arabic speakers, *n* = 10). We inserted these attributes into a self‐developed sampling matrix to constantly review which participants were still missing. Besides the fact that Arabic speakers represent a large and a growing proportion of recent migrants to Germany, we concentrated on this group to enable a more detailed analysis of potential differences due to sociocultural backgrounds for one specific, exemplary subgroup.

### 2.2 Recruitment process and channels

As COVID‐19 limited the opportunities for using ‘classic’ recruitment channels such as doctor's offices, we concentrated on alternatives. A focus here was on contacting multipliers, that is, facilities, institutions and individuals with regular and trustworthy contact with our target group, particularly (public and private) family centres, municipal facilities and kindergartens. For most of these, we forwarded the study call electronically and asked those contacts to pass it on. In addition, we used our own and partner projects' websites and social media accounts, mostly Twitter, and asked each recruited parent to forward the call to their peers. To enrol Arabic speakers, we additionally used in‐person recruitment combined with a written study invitation in specific settings. There was no affiliation with any of the research participants before this study, with no personal relationship before or during the study. And interaction before the study commencement was used to clarify the purpose of the study and participation requirements.

### 2.3 Data collection

Qualitative data were gathered via individual telephone interviews to allow participation from different regions and despite the lasting contact restrictions. This also seemed appropriate for those who may not feel confident speaking in a group discussion. A male researcher (PhD) (J. L.), and a female Arabic‐speaking researcher (Master's degree) (H. A.) experienced in qualitative research methods conducted a total of 30 interviews from August to October 2020. The average interview lasted 43 min, each one only ended once participants stated they had sufficient opportunity to elaborate on each question and could also address any further issues in a final open question. No interviews were repeated and all participants completed the interview. We offered several options to participants to avoid any interview fatigue, for example, scheduling the interview to each individual's time preferences, pausing the interview for a break to take care of the child, and conducting the interview from home via telephone or video. Participants received a 30 Euro honorarium. We developed a semistructured interview guide based on (a) the research themes and objectives, (b) the main project underlying this study^23^ and (c) core dimensions of the concept of HL. A draft version was revised by four project staff members and pilot tested with parents (*n* = 3) (Supporting Information: Appendix 2). The researchers explored participants' experiences without advanced fixed assumptions and made notes of relevant responses when possible.

We also set up a short online survey (SocSciSurvey GmbH, Germany) to further characterize the participants in terms of sociodemographic characteristics and HL (HLS‐EU‐Q16).^24^ The interview guide and the online survey were translated into Arabic (H. A.) for respective participants. To follow up on parents' IB and needs as the pandemic continued—particularly to explore changes compared to parents' statements reported here, follow‐up interviews took place from late 2021 to early 2022 and will be reported at a later stage.

### 2.4 Data analysis

All interviews were audio‐recorded, pseudonymized and transcribed verbatim (in both German and Arabic) by all project staff members using MS Word and MAXQDA (VERBI, Version 2020), without a subsequent review by the participants. Arabic transcripts were translated into English by the researcher who conducted the interviews with Arabic speakers (H. A.). We applied established principles of structuring qualitative content analysis (QCA)^25^ and used MAXQDA. QCA is common for the analysis of qualitative data^26^ and fits the purpose of a simple, in‐depth description of both variation and significant common data patterns.^27^, ^28^ Structuring QCA is considered the core of QCA,^29^ in which the material is structured based on two dimensions: cases ‘interviewees’ and categories ‘themes’, and a category system is developed.^25^ In Step 1 (pre‐analysis), we deductively coded 10 random interviews (J. L.), using the research objectives and interview guide to develop a first, broad structure of main and subcategories. In Step 2 (test phase), we applied the deductively formed Level 1 and Level 2 categories to another three interviews, analysed in full and independently by two researchers (J. L. and H. A.), inductively added Level 3 categories, and in parallel confirmed the definition of each Levels 1 and 2 categories. In Step 3 (test phase), we compared the results from Step 2, discussed the definitions and flagged up principal dissimilarities. In Step 4 (test phase), we adapted the coding scheme based on the discussions from Step 3, and agreed a final version with a third female researcher (PhD) (M.‐L. D.). In Step 5 (analysis), each researcher (J. L. and H. A.) analysed 15 interviews independently using the final categories from the test phase; additional codes were occasionally added. In Step 6 (integration), we integrated all interviews (*n* = 30) into one coding scheme, discussed new questions and unclear items and combined some similar (mostly Level 3) codes. Finally, we performed a category‐based analysis, describing and summarizing the Levels 1 and 2 categories.^26^ The results of the analysis were discussed with the author team, but not with the study participants. Initial results were, however, discussed with the study's parent panel.

To analyse the short survey, we entered *n* = 30 data sets into SPSS for a descriptive portrayal of interviewee characteristics (sociodemographic data). Using the HLS‐EU‐Q16, study participants indicated their ability to find, understand, evaluate and apply HI on a four‐point response scale (1 = very difficult, 2 = fairly difficult, 3 = fairly easy, 4 = very easy), in addition to a ‘don't know’ item. A total HL score was calculated to build three levels of HL (inadequate HL: 0–8 points, problematic HL: 9–12 points, sufficient HL: 13–16 points).^24^, ^30^

## 3 RESULTS

### 3.1 Participant characteristics

We interviewed *n* = 30 mothers and fathers, of whom *n* = 20 were German speakers and *n* = 10 were Arabic speakers. The sociodemographic characteristics and the levels of HL of the sample are illustrated in Table 1.

**Table 1:** Participant characteristics (*n* = 30)

Characteristic	*N*	%
Gender		
Male	8	26.7
Female	22	73.3
Age of parents, mean (SD) = 34.23 (5.75), range = 22–48		
18–29	5	16.7
30–39	21	70
40–50	4	13.3
No. of children, mean (SD) = 2.03 (1.098), range = 1–6		
1	11	36.7
2	10	33.3
3	8	26.7
>3	1	3.3
Age of children		
<1	3	10
1	8	26.7
2	11	36.7
3	10	33.3
≥4	14	46.7
Mother language		
German	13	43.3
Other than German	17	56.6
(School) Education^a^		
Low	5	16.7
Middle	13	43.3
High	12	30
Health literacy levels		
Inadequate (0–8)	4	13.3
Problematic (9–12)	12	40
Sufficient (13–16)	14	46.7

^a^ Education level: low: the completion of the Volks/Hauptschulabschluss/8th/9th in the German system (GE) or the elementary school education (6th class) in the Arab system (AR); middle: mittlere Reife/10th class (GE) or secondary school/9th class (AR); high: all other degrees from Fachhochschulreife, Abitur/≥12 class (GE) or high school degree/≥12 class (AR).

### 3.2 QCA

Parents' perspectives are presented here according to the main themes and subcategories derived inductively and deductively from the interviews (Table 2).

**Table 2:** Main themes and subcategories

Main theme	Subcategories
(1) Accessing and obtaining information	
Information Gathering	Information behaviour Information sources Information reasons
(2) Understanding and appraising information	
Information Handling	Positive perception of information Negative perception of information
Trust	General aspects Family, friends Classic, mass media Online media Medical personnel Other actors
(3) Applying information	
Information Handling	Processing, using of Information
Rules and Recommendations	Applying rules and recommendations
(4) Information needs and preferences	
Future Information Needs	Needs for digital offer Other needs

#### 3.2.1 Accessing (and obtaining) information

##### IB

While some parents indicated actively searching for COVID‐19–related (child) HI, others received information passively.^31^ Some did both. Over the course of the pandemic, some parents with different educational levels changed from active to passive searching or even gave up consuming information, primarily because of feeling overwhelmed, or because they did not expect new insights.

In the meantime, I take no more information, because of course I have now taken what I could take, and my head is also totally full of it. (GE, middle edu., P6:62)

##### Information sources

The most frequently used source was Google (*n* = 19), whereas Arabic speakers mentioned Facebook most frequently (*n* = 8). The latter included nonofficial sources (e.g., YouTubers, community groups, people's stories) and official, that is, public sources such as the German Ministry of Health, or HPs (doctors, scientists). The latter, however, were mostly mentioned by those with higher education. Public institutions' websites, such as the local health agency ‘Gesundheitsamt’ were the third most popular source by German speakers (*n* = 9, middle–high education). Additionally, parents frequently received information from family members, friends and acquaintances (*n* = 18), of which some had a professional healthcare background. Regarding online sources, Arabic speakers mentioned YouTube in particular, whereas German speakers referred to Podcasts (e.g., NDR Corona update). Other popular sources for Arabic speakers included childcare facilities (e.g., kindergartens), whereas German speakers rather cited medical experts, including relatives and friends. The former were mostly seeking, reading or hearing information in Arabic (online and offline), due to their limited German language proficiency. They preferred information in their native language, to ease understanding and save time.

For me here in Germany, these YouTubers were coming out (mentions names) I was following them, they were saying daily the recovered and infected cases and advising […], and they are not a public source, I mean a private source […]. (AR, middle edu., P3:51–52)

##### Information reasons

Most parents (*n* = 22) reported searching for general COVID‐19 information, that is, about the transmission pathways. This contrasts with child‐specific information such as infection risks, but also nonhealth‐related aspects such as contact restrictions imposed protectively by childcare facilities. Child‐related information was of particular interest to pregnant women, parents with a chronically ill child or visiting a childcare facility. Other important topics included infection and safety measures, the number of infected and death cases, and high‐risk groups. Parents also reported seeking others' opinions about and experiences of the disease. When seeking additional information, German speakers primarily referred to federal state requirements, hospital measures and a future COVID‐19 vaccine, whereas Arabic speakers rather referred to dietary intake and information concerning countries in the Arab region.

I informed myself about the children at the very beginning and it was said that things would be milder and I didn't think about it that much then. I've been looking more for the numbers, how many people are getting infected, is it going up or down and so on and what the signs are, how it's happening […]. (GE, low edu., P13:22)

#### 3.2.2 Understanding and appraising information

##### Information perception

Some parents found the available information helpful and sufficient to protect their families, and referred to it as being consistent, accurate, detailed and easily accessible, particularly online. However, many expressed facing uncertain, confusing or contradictory information, and reported being exposed to a great deal of false information. Some parents repeatedly considered information to be dramatizing and inaccurate—particularly information found via Google—whereas some found it to be scientific in nature, leading to difficulties in comprehension.

Yes, even now I have seen a few reports in YouTube. There are virologists who say something and then there are virologists who say something else […] What is true now? You're really confused again because when you hear opinions from experts, you can't really believe them either. (GE‐middle edu., P18:44)

##### Trust

While few parents indicated a general level of trust, many reported that, first, a clear decision on trust is difficult to make, particularly because of the variety of (online) sources. To determine trustworthiness, parents repeatedly mentioned checking and comparing multiple sources with their previous knowledge or personal perception. Others, mostly German speakers (middle–high education) differentiated between the sources and specified several criteria for trust, particularly regarding the author (identity, qualifications, seriousness, neutrality).

Second, trust depends on the source: It was mostly ascribed to nondigital sources, particularly medical personnel, given their professional knowledge and expertise. Though parents used a range of information sources, they stressed that paediatricians and family physicians are the first and most reliable references, and said they followed their advice. Only a very few mentions relate to not relying on a paediatrician for pandemic‐related information and to the necessity for a second, specialist opinion.

The primary 100% trusted source is the doctor. (AR, low edu., P9, Pos. 42)

So I would say that if it was about my children, if I noticed that they had any symptoms or pain, then I would honestly say that I would not trust anyone except the doctor. (GE, low edu., P20:56)

Trust also seemed to be comparatively high for Arabic speakers in kindergartens and schools, as they apply the rules and recommendations to protect children. In comparison, German speakers rather referred to governmental public health (RKI, Gesundheitsamt), and scientific, that is, medical institutions (medical schools, health experts, journals' studies, health magazines (e.g., Apothekenumschau). Few reported scepticism of public institutions (e.g., World Health Organization), the pharmaceutical industry, health experts and funded scientific studies, for example, due to inconsistency.

Regarding family members and friends, parents varied in their appraisal of trust, and ascribed most trust to those relatives and friends who had medical knowledge or experience of the disease. In cases of distrust, this was due to perceiving advice as subjective, influenced by emotions or nontransferable to one's own situation.

Varying perceptions of trust were also found for online sources, which were distrusted by Arabic and German speakers because of its multiple conflicting opinions. Parents also stated that the internet is an open place, where those with no knowledge or expertise share information, including nonfactual, and some parents found it difficult to identify the information producer or its reliability. In addition, parents criticized using Google for disease diagnosis, as this causes fear from search results. However, Arabic speakers especially trusted online sources (*n* = 7) when this was provided by doctors on social media, or Google, or by previously known YouTubers. These convey or translate information in Arabic, which guarantees understanding, in addition to providing references for delivered information, which allows parents to check its validity. Arabic speakers more often ascribed distrust to classic media, for example, given a lack of transparency about infection statistics.

#### 3.2.3 Applying information

##### Information processing and use

While some parents were overwhelmed and affected by false and negative information, many expressed that it did not affect them. Few said it was easy to identify fake news based on personal judgement, common sense or replicability of information from multiple sources. A few argued with others about veracity (e.g., of conspiracy theories) and tried to convey correct information to them.

When deciding which information to use, parents implemented different approaches: deliberately selecting the quantity and/or kind of relevant information (for child health); matching it to their current situation; weighing multiple sources against each other or against previous knowledge; discussing information with others (e.g., family, peers, colleagues), and exchanging opinions, experiences and knowledge; applying reasoning, common sense or relying on gut instinct or personal judgement; collecting information from (multiple) digital sources to get an initial idea about the subject and, following this, consulting a doctor to avoid having to make their own decision.

So it's always been different and then I was just a little bit like what am I doing now and then somehow you acted according to your gut feeling. (GE, middle edu., P9:50)

Few parents reported looking for more, detailed information, for example, to gain more relevant, specific or additional knowledge. Regarding others' opinions and experiences, some indicated that these influenced their own opinion and information decisions. Others found such experiences inapplicable. Furthermore, some parents reported educating children about the disease.

##### Applying rules and recommendations

In terms of committing to public health safeguarding measures, most parents (*n* = 27) reported following and applying these in their daily life, especially at the beginning of the outbreak. As the pandemic continued, nearly a third reported to still adhere strictly, whereas others reported lower adherence and easing, for example, in settings where in‐person contact with relatives occurred as restrictions were eased. Additionally, some indicated that their own or others' (family or friends) disease experience affected their adherence and precaution, as they undergo or learn about the, most often, mild course of the disease. Parents also stressed the difficulty of strictly following the restrictions regarding social contacts and safe physical distancing, for example, in playgrounds, where there is inevitable interaction. Sometimes, this led to not continuing or becoming tired of applying some guidelines in daily situations with the child.

[…] there are many things not like before […] one wears a mask, I told you there is no shaking hands, we try to keep distance but there are things involuntary for example, we want to eat together at the same table, what can you do? Nothing. So, it is not negligence but there is easing. (AR, high edu., P8:79–80)

#### 3.2.4 Information needs and preferences

First, parents referred to how HI is communicated (communicating, messaging), pointing out that dissemination should happen through kindergartens and schools, particularly to reach migrant parents, besides general public information campaigns via classic broadcast and print media.

[…] I think most families are actually reached via television and radio and especially posters. […] just pictorial and large and appeals to everyone […]. (GE, high edu., P7:96–98)

Parents stressed the role of frequently accessing HPs, particularly paediatricians (midwives were less relevant for Arabic speakers), regarding emerging disease knowledge and its impact on children's health, receiving instructions and advice in daycare centres and finding and applying child health‐specific information. While parents did want to understand whether COVID‐19 is a threat for children and if this is based on evidence, they also desired COVID‐19 to be treated as a ‘normal’ disease to avoid further panic, particularly for new parents, and instead focus on advice that is helpful for dealing with the crisis situation more generally, for example, regarding nutrition, mental hygiene and social contact.

Compared to nondigital sources, it seemed rather difficult for parents to state clear preferences for digital information; they repeatedly mentioned not feeling the need for specific changes, or feeling well‐informed. A few mentions related to increasing the transparency and up‐to‐dateness of digital information and its respective sources—preferably public sources—and adding options for direct, personal interaction with HPs in case of specific questions. Arabic speakers desired audio‐visual and translation tools to facilitate understanding, as this would be beneficial for saving time.

[…] When the information is issued from an official authority, it is more reliable than the doctor because it is issued by an official body, meaning it targets all people. Yes, sure it is much better to be in Arabic but I tell you again, even when the information is issued by a responsible authority and only in German, [it is important that] there is someone who translates the information and passes it on to us […]. (AR, high edu., P1:130–132)

Parents also referred to the communication of public health messages, for which a few Arabic speakers mentioned the need to raise awareness about the necessity of adhering to guidelines. Further, they urged the need for better disease control and management of rules and guidelines, particularly in private settings and for regularly experienced situations, for example, (crowded) childcare facilities. In that sense, the need for better coordination among parents and childcare facilities (here: kindergartens) by educating staff to deal wisely with COVID‐19–related information and making child‐related decisions, such as deciding whether a child with symptoms should stay at home, was also mentioned. German speakers called for rules and regulations to be issued and applied uniformly nationwide.

## 4 DISCUSSION

### 4.1 Information sources

Parents use both formal and informal sources for pandemic‐related information, predominantly online. Existing research outlined the increasing use of online sources for health and medical information^32^ and its importance during the COVID‐19 outbreak.^33^ In Germany, the internet is the fourth most important HI source, in general, after mass media, HPs and family members.^34^ In our study, parents frequently googled COVID‐19, whereas migrant parents more often relied on social media platforms (SMPs), finding them easier to access, more up‐to‐date^35^ and available in their native language, which helped them to understand and practice preventive measures. The reliance of migrants with limited local language skills on (informal) media channels has been reported in Oktavianus et al.^19^ Recent research highlights the reach of information delivered through SMPs to diverse population groups and its role in promoting health prevention behaviour. Nevertheless, it underscores the vast spread of mis‐ and disinformation and its potential harm to health.^32^ Official information sources were more used by German speakers, mostly those with middle–high education, while only few, high‐educated Arabic speakers named (inter‐)national institutions, almost without referring to other institutional information channels responsible for crisis communication.^36^ This is probably due to limited language proficiency and lack of knowledge of the German health systems' communication channels, according to Finell et al.^37^ As Arab parents often relied on accessible and familiar information sources, there could be a focus on engaging more informal information mediators for these groups, as suggested by Mason et al.^38^

#### 4.1.1 IB

Over the course of the pandemic, many changed their IB, and only a few remained active information seekers. Griebler et al.^36^ show a reduction of interest in and need for COVID‐19‐related information and/or ‘selective usage behaviour’ among the Austrian population. At the pandemic's onset, uncertainty and perceived seriousness of the disease for their children's health triggered more active IB, which may be due to risk perception and uncertainty as drivers for seeking COVID‐19‐related information, as expressed by Huang and Yang.^39^ However, the evolving knowledge, its effect on children and the stream of ample information led to a change in the state of emergency and the need to constantly seek related information, particularly when either feeling overwhelmed or satisfied.^19^ Although almost all parents sought information on infection prevention, our study highlights a slight difference between the two groups regarding other issues of interest (e.g., requirements of federal states vs. state of infection in (Arab) home countries). Here, Oktavianus et al. point at migrants' information due to concerns about the safety of family members in their home country, not only for their own safety. Hence, parents may be in a dual or even triple role of seeking information for themselves, for their child, and for further family members.^19^

#### 4.1.2 Information handling

Though parents were aware of the infodemic, the overflow of (false and misleading) information still led to confusion and uncertainty. The susceptibility to the infodemic might be partly explained by reliance on, for example, SMPs, especially by Arabic speakers, facilitating the dissemination of misinformation.^40^ However, the results indicate that it did not constitute a problem for some parents who reported not encountering, ignoring or avoiding such information. Tandoc et al.^41^ stated that readers often ignore fake news, but in some cases may act on it. Moreover, some parents felt able to distinguish correct from incorrect information; though to do that they relied on their own judgement.^42^ This is in line with the internal and external authentication of Oktavianus et al.^19^ Prior research explained the use of nonrational factors for judging information when there is a lack of knowledge and conflict among information sources, emphasizing the need for critical thinking to handle misinformation.^42^ Here, consideration should be given particularly in the case of migrant parents, as previous research points at the difficulties of knowing which (reliable) sources to turn to when not being familiar with the HI context in a different country.^37^

Okan et al.^43^ outlined the continuous and broad provision of coronavirus prevention measures through various channels that were easy to understand and apply, and their positive effects on people's HL. This concurs generally with the parents' perception of the usefulness of available COVID‐19 information, and is particularly obvious for migrant parents, due to the ease of access to different, largely online sources in Arabic. Additionally, this is vital for newcomers who may especially lack social interaction and support in the host community, particularly amid the imposed restrictions.^44^ In addition, our study indicates that digitally seeking COVID‐19‐specific HI seemed helpful for parents in making child health decisions during the outbreak. However, it shows that the physician is the main, if not the primary, reference to consult in terms of child health issues or to verify information, which resonates with previous findings by Jaks et al.^45^

Our study shows that parental adherence to PH measures is influenced by: the context and nature of the activity (e.g., indoor vs. outdoor); the compliance, acceptance and support from the social environment (private or public); the consistency of the issued rules and guidelines; the organization and management of resources (e.g., public transportation) and the personal beliefs, experiences and mentality. Oktavianus et al.^19^ reported similar findings regarding the effect of external factors on adopting preventive behaviours during the outbreak. This corresponds to what King et al.^46^ underlined regarding the influence of social and structural—in addition to individual determinants of health—on compliance behaviour. Moreover, Benham et al.^47^ referred to the Theoretical Domains Framework of Atkins et al.^48^ to explain ‘the need to understand the characteristics of the people in whom a change is to be effected, their behavioural context, and the components driving change, in order to facilitate behaviour change’.

#### 4.1.3 Trust

Parents trusted medical personnel the most, confirming previous research,^49^ and also in terms of COVID‐19 information.^36^, ^50^ In our study, they do not fully trust online sources, including SMPs; trust varied depending on the sources used. Fewer found classic media sources (highly) reliable and trustworthy for COVID‐19 and rather a source of fear, which is supported by Finell et al.^37^ Griebler et al.^36^ observed a loss of trust in TV, radio, internet and health authorities as the pandemic evolved. Though some parents are acquainted with some ‘quality criteria’, we found that parents may not consciously apply them. This is in line with Slomian et al.,^51^ in which very few participant women seemed to be aware of the existence of any quality standards for HI sites. Looking at the sociocultural diversity of target groups, there could be further research to understand if trust can be better established by optimizing the use of quality criteria, or whether the focus should be on engaging professionals and institutions that guide, for instance, migrant parents to the ‘right’, that is, high‐quality sources. In particular, Bergman et al.^52^ highlight the need for tailored, effective strategies as well as the importance of social (information) networks.

Further, others' opinions and experiences are not applicable for some parents to their own situation. However, some emphasized that exchanging information and knowledge with family and friends, and learning about shared stories on SMPs was a reason for trusting information obtained from peers with experience of the disease. Scholars referred to these experiences as ‘testimonies’, ‘case reports’^34^ or ‘experiential knowledge’ and related it to ‘social support’^42^ that serves to support decision‐making and understandability.^34^

#### 4.1.4 Needs and preferences

Parents required online sources to satisfy their information needs, though these do not replace direct personal interaction with HPs. Previous research has shown that digital media complements rather than substitutes for traditional HI sources.^45^ Further, our study underscores that other means of communication are important to parents, endorsing the significance of considering multiple information channels. Our findings emphasize the importance of HI being comprehensible, navigable and official. This aligns with the suggested principles for designing HI in prior research.^32^ Additionally, we shed light on the preference of participants from other cultural backgrounds to receive information in their native language. This confirms previous calls to constantly provide reliable information and help in migrants' native language, or more generally, in multiple languages.^37^, ^53^, ^54^, ^55^, ^56^ Our results also support the finding of existing research that ‘high‐quality HI must be (…) culturally competent—the ability to interact effectively with diverse audiences by recognizing and responding to variations in social, cultural, and linguistic needs’.^32^ Hence, while previous research pointed to the Internet to spread culturally sensitive HI,^52^ our findings suggest that respective sources need to be provided by those deemed as trusted and familiar within a specific community, for example, (Arabic) medical experts on YouTube or Instagram. When developing respective approaches, ‘cultural mediators’ should be included.^57^ In terms of format preferences of digital information, which may affect parents' understanding and evaluation, the use of visual and interactive communication tools that may also allow (online) contact with HPs, display elements (e.g., bulleted points) and use of simple language are in line with previous findings.^34^, ^53^, ^58^

### 4.2 Study contributions and future research

The current study contributes to the understanding of IB, how parents make decisions and apply information to care for their child, and what they need to do so in a pandemic situation. For future research, it is essential to study IB and the role of trust in specific sources on information and prevention decisions at different time intervals, given the changes in IB over time Additionally, observing the actual (digital) search behaviour may yield a better understanding of parents' considerations. We also explored differences among diverse parent groups, and further assessments using quantitative or structured qualitative survey studies seem warranted, particularly to specify support needs for parents who are unfamiliar with the host countries' information channels and providers. Future research could also assess parental IB for noncrisis health situations, where information may be more difficult to access.

### 4.3 Limitations

The interview and survey translation was done by an Arabic‐speaking researcher. While this does not guarantee comprehension and validity, research suggests that the adaptation of the English version of HLS‐EU‐Q16 is applicable to the Arabic language, to measure HL among native Arabic‐speaking people in Sweden.^52^ Likewise, the verbal translation from Arabic to English represented a challenge to ensure the meaning or stay true to the interviewee narratives. Besides that this researcher is a native Arabic speaker, hence reducing the chance of misunderstandings, the focus was on avoiding interpretations, that is, translating expressions that may contain different meanings in its most literal sense. Since our sample size was predetermined and we applied purposive sampling, we focused on ensuring sufficiently diverse participant characteristics using a sampling matrix. The nonrandomized selection implies subjectivity,^59^ but qualitative research and purposive sampling do not aim generalization. The over‐representation of mothers (*n* = 22) may imply that fathers' views were not fully captured. Furthermore, parents with a low educational level are underrepresented (*n* = 5), which points to the difficulty of accessing this group. Lastly, while we could not (fully) achieve transcript validation through participant feedback, initial results were discussed with the established parent panel to promote research validity.

## 5 CONCLUSION

Overall, the spread of pandemic‐related information, specifically on infection protection and safety measures, through a multitude of channels allowed parents to access and select sources that satisfy their information needs. However, the results reveal some differences between the two groups regarding the type of information sources preferred, and their knowledge of the health (information) system, and of the attributes of high‐quality HI and credible (digital) sources. This has critical implications for the type and quality of information content, thus influencing (parents') beliefs and decisions to act preventively. It is a joint responsibility between HPs and information producers to empower parents to make informed health decisions for their children and to point at strategies for identifying dis‐ and misinformation. Besides acting as a primary source, especially for parents who encounter difficulties appraising and using digital information, HPs are key to give guidance about high‐quality (digital) information sources. This prompts engaging HPs in crisis communication and training them on delivering culturally competent information. It is imperative for information producers to provide parents with adequate information about the current state of knowledge, use multiple communication channels and pay attention to differences among distinct groups. This requires the involvement of parents' representatives, including those from migrant communities, in the development of communication strategies for future health crises. Public health initiatives should foster reliable information sources, build trust in these sources and educate parents, particularly (new) immigrants, to navigate the health (information) system, thereby fostering their HL.

## AUTHOR CONTRIBUTIONS

**Hala Altawil**: Methodology; investigation; writing – original draft. **Ronny Klawunn**: Methodology; writing – review and editing. **Marie‐Luise Dierks**: Conceptualization; writing – review and editing; supervision; funding acquisition. **Jonas Lander**: Conceptualization; methodology; investigation; writing – review and editing.

## CONFLICT OF INTEREST

The authors declare no conflict of interest.

## ETHICS STATEMENT

The Ethics Committee of Hannover Medical School approved this study (ID 8161_BO_K_2018_Extension2020). Written consent was obtained from all study participants in accordance with ethics approval. Participation was voluntary. Financial incentives for participation were offered.

## Supporting information

Supporting information.

## Data Availability

The data presented in this study are available on request from the corresponding author. The data are not publicly available due to the need to preserve the anonymity of the interview partners.
