# Supplementary material for: Parental COVID‐19–related health information practises, sources, evaluations and needs: A qualitative interview study
Source: Health Expect. 2022 Dec 8;26(1):555–65. doi: 10.1111/hex.13688 (PMC9854324; doi:10.1111/hex.13688)
Supplement: Supplementary file 1 — Supporting information. [file HEX-26-555-s001.docx]

**Appendix 1. Consolidated criteria for reporting qualitative studies (COREQ): 32-item checklist**

Developed from:

Tong A, Sainsbury P, Craig J. Consolidated criteria for reporting qualitative research (COREQ): a 32-item checklist for interviews and focus groups. *Int J Qual Health Care*. 2007;19(6):349-357.

| **No. Item** | **Guide questions/description** | **Reported on page #** |
| --- | --- | --- |
| **Domain 1: Research team and reﬂexivity** | | |
| *Personal Characteristics* | | |
| 1. Interviewer/facilitator | Which author/s conducted the interview or focus group? | 3 (Materials and Methods) |
| 2. Credentials | What were the researcher’s credentials? E.g. PhD, MD | Title page |
| 3. Occupation | What was their occupation at the time of the study? | 3 (Materials and Methods) |
| 4. Gender | Was the researcher male or female? | 3 (Materials and Methods) |
| 5. Experience and training | What experience or training did the researcher have? | 3 (Materials and Methods) |
| *Relationship with participants* | | |
| 6. Relationship established | Was a relationship established prior to study commencement? | 3 (Materials and Methods) |
| 7. Participant knowledge of the interviewer | What did the participants know about the researcher? e.g. personal goals, reasons for doing the research | 4 (Materials and Methods) |
| 8. Interviewer characteristics | What characteristics were reported about the interviewer/facilitator? e.g. Bias, assumptions, reasons, and interests in the research topic | n.a |
| **Domain 2: Study design** | | |
| *Theoretical framework* | | |
| 9. Methodological orientation and Theory | What methodological orientation was stated to underpin the study? e.g. grounded theory, discourse analysis, ethnography, phenomenology, content analysis | 4 (Materials and Methods) |
| *Participant selection* | | |
| 10. Sampling | How were participants selected? e.g. purposive, convenience, consecutive, snowball | 3 (Materials and Methods) |
| 11. Method of approach | How were participants approached? e.g. face-to-face, telephone, mail, email | 3 (Materials and Methods) |
| 12. Sample size | How many participants were in the study? | 3 (Materials and Methods) |
| 13. Non-participation | How many people refused to participate or dropped out? Reasons? | n.a |
| *Setting* | | |
| 14. Setting of data collection | Where was the data collected? e.g. home, clinic, workplace | 3 (Materials and Methods) |
| 15. Presence of non-participants | Was anyone else present besides the participants and researchers? | 3 (Materials and Methods) |
| 16. Description of sample | What are the important characteristics of the sample? e.g. demographic data, date | 4-5 (Results) |
| *Data collection* | | |
| 17. Interview guide | Were questions, prompts, guides provided by the authors? Was it pilot tested? | 3-4 (Materials and Methods) |
| 18. Repeat interviews | Were repeat interviews carried out? If yes, how many? | 3 (Materials and Methods) |
| 19. Audio/visual recording | Did the research use audio or visual recording to collect the data? | 4 (Materials and Methods) |
| 20. Field notes | Were ﬁeld notes made during and/or after the interview or focus group? | 4 (Materials and Methods) |
| 21. Duration | What was the duration of the interviews or focus group? | 3 (Materials and Methods) |
| 22. Data saturation | Was data saturation discussed? | 13 (Discussion) |
| 23. Transcripts returned | Were transcripts returned to participants for comment and/or correction? | 4 (Materials and Methods) |
| **Domain 3: Analysis and ﬁndings** | | |
| *Data analysis* | | |
| 24. Number of data coders | How many data coders coded the data? | 4 (Materials and Methods) |
| 25. Description of the coding tree | Did authors provide a description of the coding tree? | 4 (Materials and Methods) |
| 26. Derivation of themes | Were themes identiﬁed in advance or derived from the data? | 4 (Materials and Methods) |
| 27. Software | What software, if applicable, was used to manage the data? | 4 (Materials and Methods) |
| 28. Participant checking | Did participants provide feedback on the ﬁndings? | PPI statement (Abstract) |
| *Reporting* | | |
| 29. Quotations presented | Were participant quotations presented to illustrate the themes/ﬁndings? Was each quotation identiﬁed? e.g. participant number | 5-9 (Results) |
| 30. Data and ﬁndings consistent | Was there consistency between the data presented and the ﬁndings? | 4-12 (Results-Discussion) |
| 31. Clarity of major themes | Were major themes clearly presented in the ﬁndings? | 5-10 (Results) |
| 32. Clarity of minor themes | Is there a description of diverse cases or discussion of minor themes? | 5-10 (Results) |

**Appendix 2. Interview Guide**

Interview guide for parents on COVID-19 and child health

*Topics/questions:*

*Greeting, organizational matters*

*Introduction*

COVID-19 has had a major impact on our lives for several months. In our research project, we asked ourselves what the situation is like for parents at the moment, precisely because you also take care of the family and especially the children.

*General experience*

So, it would be great if you tell us how you as a mother/father have experienced COVID-19 so far. And first of all, of course: how many children live in your household and what are their ages?

1. How was the situation for you and the children in the last few months, or in other words: Have things changed in your everyday life (with your family)?
2. If yes: how, what, and what are the consequences for you and your family?
3. Are you worried about your child, about yourself, about your family, and also with a view to the next few months? What are they?

*General Information Search*

As mentioned briefly at the beginning, we would also like to learn more in our study about how you search for and then deal with health information, especially with regard to your child(ren).

1. Is it more that you are actively and specifically looking for information, or could one say: you take what you hear with you? Why?
2. How have you (specifically) obtained information on the subject of “COVID-19 and children's health” so far?
3. With whom did you talk about it?
4. What questions and topics were it about?
5. Were you concerned with child health or just COVID-19 as a whole?
6. Who or what is your “primary” source of information? (Internet, pediatrician, TV, radio, etc.)
7. Can you roughly describe how you look for information or when and how you hear something about child health and COVID-19, talk about it, etc.?

Perhaps using an example?

Case 1: Let's assume you want to find out more about the protection of your child against COVID19 (or related topics, e.g., risks of contact with older people ...), how do you proceed or how would you proceed to find trustworthy information?

1. When there is hardly any search, discussions, etc.
2. Why not? Why only so limited?
3. What did you do instead?
4. And when you have found an answer to your question(s), how did you deal with it?
5. Have you spoken to family members, friends, or specialists about it, for example?
6. Have you been able to use the information for yourself and your family?

- If yes, how did you do it? If not, why not?
- Have you thought if this is "good" information for you, or if you want to continue searching?

1. How satisfied have you been with the offered information on COVID-19 so far? And how does that relate to child health?
2. Which “sources” do you find good, credible, and understandable?
3. Why?
4. Do you have an example of this?
5. Is there something that you are missing?

*Dealing with information*

*Information overload*

1. Especially at the beginning of Corona and also now it is often the case that there is a lot of information, even on the same question. How have you experienced that so far?
2. Is this a problem for you? Why? Why not?
3. Do you have an example of this?
4. Case 2: Let's assume that your child is now old enough that s/he should go to kindergarten in September, and you now want to find out how you can protect your child there and what’s the "right" thing to do. When searching, you will find a whole range of different options and opinions.

Alternatively, more general: "... what should you pay attention to in the next few

months, and when it comes to autumn/winter in order to protect the child"

1. What do you do then?
2. How do you deal with it?

*Misinformation*

1. Among all this, there is often a great deal of information - did you have the impression that things were written or said that were not correct?
2. If yes, what example? Where? How did you deal with it? Was that a problem for you?
3. If not, have you specifically avoided false information, or did you have the impression that it does not occur with COVID-19 at the moment? How? Why?
4. How do you determine that information is false? How do you go about that?

*Fake news*

1. Was the subject of fake news something that you also came across?
2. Did you deal with it in any way? (e.g., Trump statements, children who are exploited for COVID-19 therapy development, useless masks, conspiracy theories)
3. Why did (not) that play a role for you?

*Overall*

1. Do you see the subject of information overload, misinformation, and fake news “only” in relation to COVID-19 in general, or also specifically in relation to information on child health? How far?
2. How satisfied are you with the amount and quality of information when it comes to COVID-19 and child health?
3. What could be better? What would you personally wish for?
4. Would you like to receive or hear less new information, new conditions, rules, etc. or is that not a problem?

*Influence of trust on information behaviour and decision-making processes*

We are also interested in the topic of “trust in information and the influence on one's own

actions”.

1. What would you say, to what extent do you trust the information you find about

COVID-19 and child health so far?

1. Can you describe what affects your trust in information? Are there certain characteristics that make the information trustworthy? What are these?
2. Who or what do you generally consider (not) trustworthy when it comes to COVID-19? (Doctors? Midwives? Family? Relatives, friends? TV, radio? Magazines? Internet? etc.?)
3. Why is there a difference for you here?
4. If you think specifically about doctors and midwives:
5. How well informed do you feel? Are these people good consultants?
6. Have you talked about Covid-19?
7. Would you like more consultation?
8. How could you better support doctors or midwives with information and decision-making?

*Acceptance and implementation of (behavioural) guidelines and recommendations*

Back to the question at the beginning, how do you implement all the recommendations, regulations, and so on…

1. How easy was it for you at the beginning of the pandemic to implement the rules in everyday life, especially with regard to your child(ren)?
2. Have you experienced situations in which you could not implement information?
3. Was it the situation or was the information not practical?
4. And now, has anything changed?
5. What about contacts with other families and friends, for example? Are you doing this again? Why yes /no?
6. Do you talk to others, e.g., friends, how do they do it?
7. Do you have the feeling that you are consistently implementing everything that is important, or do you find that you are careless?
8. Does the internet play a role for you in being able to implement health information effectively (in your family, in everyday life?)
9. What would help you to find and use health information better?

*Future situation and conclusion*

In the last few months, we have been through a phase with a lot of concern and restrictions, and so far no one really knows how things will be in the next 2-5 months.

1. How do you see the next few weeks, and months for your family, and your child?
2. Do you have certain worries or fears?
3. Are you afraid of infection?
4. Do you think you are (too) afraid of Corona?
5. Does this assumption apply specifically to your child or to most others as well?
6. Is there anything else you have already thought about?
7. I still have three final questions:
8. Do you think that you will inform yourself more about COVID-19 in the future than you did before? Why yes /no?
9. Is there anything that makes you skeptical or even worried when looking for information?
10. Do you see the internet in particular as support for your questions and points?
